# Supplementary material for: FST and genetic diversity in an island model with background selection
Source: PLoS Genet. 2024 Dec 2;20(12):e1011225. doi: 10.1371/journal.pgen.1011225 (PMC11637402; doi:10.1371/journal.pgen.1011225)
Supplement: S1 Appendix — (PDF) [file pgen.1011225.s001.pdf]

F_{ST} with a finite number of demes and migration immediately preceding measurement

Our simulations in SLiM involved a finite number of demes in an island model with drift preceding migration followed by assessment of the value of F_{ST}. The effect of a finite number of demes has been assessed by several authors (see Ref. [32]); and having migration immediately precede assessment has been shown to reduce F_{ST} by Sved and Latter [48], albeit in a model with an infinitely large number of demes. Here we show calculations to predict F_{ST} of neutral loci accounting for both of these factors.

Here we will follow Slatkin [33], who showed that F_{ST} could be calculated as a function of the probability of nucleotide differences of two alleles chosen from the same sub-population (π_s) or from two separate sub-populations (π_B) in a finite island model with d demes. Slatkin [33] defined F_{ST} using the probability of sequence differences of two randomly chosen alleles, sampled without respect to which population they were from, or π_T, which when all demes are equal in size is given by

$$\pi_T = \frac{\pi_S}{d} + \frac{(d-1)\pi_B}{d}.$$

Slatkin's definition of F_{ST} is

$$G_{ST} = 1 - \frac{\pi_S}{\pi_T}$$

This definition of F_{ST} equivalent to what is often called G_{ST} (therefore we will refer to it as G_{ST} here to discriminate it from $F_{ST} = 1 - \pi_S/\pi_B$ that is estimated using π_B rather than π_T as the standard.)

Slatkin [33] showed that F_{ST} can be calculated from the mean coalescence times of a pair of alleles sampled both from the same population ($\overline{t_S}$) or from two separate populations ($\overline{t_B}$).

$$G_{ST} = \frac{\bar{t} - \bar{t_S}}{\bar{t}}$$

Here, $\bar{t}$ is the mean coalescent time of two alleles sampled at random without respect to which population they came from:

$$\bar{t} = \frac{\bar{t_S}}{d} + \frac{(d-1)\bar{t_S}}{d}$$

The equivalent expression for F_{ST} defined with π_B (as is implied, for example, in Weir and Cockerham's estimate of F_{ST} [31]) is

$$F_{ST} = \frac{\bar{t_B} - \bar{t_S}}{\bar{t_B}}$$

Slatkin showed that, for the case when migration precedes drift, the mean coalescent times for diploids are given by

$$\bar{t_B} = \bar{t_S} + \frac{2m - m^2}{d-1}$$

(Here we do not use the small migration approximation that Slatkin used for $\bar{t_B}$.) The latter equation calculates the time required for two alleles in different demes to have both been in the same deme in the last term, which then adds to the coalescent time of two alleles in the same deme for the final answer.

If the population is measured immediately after migration, then we can calculate the values of the mean coalescent times by modifying $\bar{t_S}$ and $\bar{t_B}$ by one episode of migration. We will use asterisks to indicate the values of the mean coalescent times calculated in this way:

$$\bar{t_S}^* = (1-m)^2 t_S + 2m(1-m)t_B + m^2\left(\frac{t_S}{d-1} + \frac{t_B(d-2)}{d-1}\right) \quad \text{(A1)}$$

and

$$\bar{t_B}^* = (1-m)^2 t_B + 2m(1-m)\left(\frac{t_S}{d-1} + \frac{t_B(d-2)}{d-1}\right) + m^2\left(\frac{t_S}{d-1} + \frac{t_B(d-2)}{d-1}\right) \quad \text{(A2)}$$

We can now find F_{ST} for this order of events:

$$F_{ST}^{*} = \frac{\overline{t_B}^{*} - \overline{t_S}^{*}}{\overline{t_B}^{*}} \quad \text{(A3)}$$

Putting equations A1 and A2 into Equation A3, we find, after some algebra:

$$F_{ST}^{*} = \frac{1}{1 + \frac{m(d-1)(d(2-m)\left(2N_{e,local}+1\right)-2)}{(d(1-m)-1)^2}}$$

Assuming $N >> 1$, we can approximate this by

$$F_{ST}^{*} = \frac{1}{1 + \frac{(d-1)d(4-2m)N_{e,local}m}{(d(1-m)-1)^2}} \quad \text{(A4)}$$

When the number of demes d is large, this recreates the result of Sved and Latter [48].

Let's turn now to predicting G_{ST} immediately after migration, or

$$G_{ST}^{*} = \frac{\bar{t}^{*} - \overline{t_S}^{*}}{\bar{t}^{*}}$$

Using the values of $\bar{t}^{*} and\ \overline{t_S}^{*}$ above we can find:

$$G_{ST}^{*} = \frac{1}{1 + \frac{dm\left(d(m-2)\left(2N_{e,local}+1\right)-2\right)}{(d(1-m)-1)^2}}$$

Again when $N >> 1$, this is well approximated by

$$G_{ST}^{*} = \frac{1}{1 + \frac{d^2 2(2-m)N_{e,local}m}{(d(1-m)-1)^2}} \quad \text{(A5)}$$

For haploids, the corresponding formula are

$$F_{ST}^{*} = \frac{1}{1 + \frac{(d-1)d(2-m)N_{e,local}m}{(d(1-m)-1)^2}} \quad \text{(A6)}$$

and

$$G_{ST}^{*} = \frac{1}{1 + \frac{d^2(2-m)N_{e,local}m}{(d(1-m)-1)^2}}. \quad \text{(A7)}$$
