# Supplementary material for: FST and genetic diversity in an island model with background selection
Source: PLoS Genet. 2024 Dec 2;20(12):e1011225. doi: 10.1371/journal.pgen.1011225 (PMC11637402; doi:10.1371/journal.pgen.1011225)
Supplement: S1 Text — (PDF) [file pgen.1011225.s002.pdf]

Derivation of B_{local} with 'migration effect' and application to the Good et al. [23] method

In this section, we first use the approach from the Appendix of Ref. [43] to account for how migration affects *B* in structured populations. Then, we incorporate our derivation into the framework of Good et al. [23] to make predictions under weak selection, in the *interference selection regime*. In a metapopulation, suppose we have an island model with *d* subpopulations, each with N_{local} diploid individuals. Deleterious mutations appear at site *i* (and eventually achieve mutation-selection balance) with per-base pair mutation rate μ_i and heterozygous selection coefficient t_i (fitness is multiplicative across sites). Recombination between neutral and deleterious sites occurs at rate r_i. Recombination changes genotype frequencies only if individuals do not die from selection following the recombination event; hence, the effective recombination rate is $r_i' = r_i(1 - t_i)$. Migration occurs at rate *m*, with *m* fraction of individuals in each deme migrating out each generation and being replaced by immigrant individuals which come from a randomly chosen other subpopulation.

In order to predict the effect of background selection on F_{ST}, we need to understand the effect of background selection on the effective population size of a single deme: the *local* effective population size, $N_{e,local}$. The local effective population size will be affected by the variance in reproductive success of neutral alleles created by their association with deleterious mutations, just as in any undivided population. However, if an individual with the deleterious mutation migrates to another deme, any subsequent effects on the reproductive success of linked neutral sites will not affect $N_{e,local}$. Therefore, we are interested in the variance in reproductive success of the neutral allele due to its association with the deleterious allele that affects the local

distribution of reproductive success. Such variance in reproductive success will influence the local effective population size, $N_{e,local}$, and ultimately the local neutral diversity, π_{local}, within the subpopulation. Hereafter, we denote the geographically local effect of background selection on individuals within a focal subpopulation as B_{local}.

There are four factors that will influence the effect of background selection on $N_{e,local}$: the deleterious mutation rate per base-pair, μ_i, the heterozygous selection coefficient, t_i, of deleterious mutations, the recombination rate between the deleterious and neutral allele, r_i, and the migration rate, m. The deleterious mutation rate, μ_i, will affect the frequency of introduction of deleterious alleles, thus directly influencing the variance in reproductive success of neutral alleles and ultimately B_{local}. The selection coefficient of a deleterious allele will influence the number of generations it remains in the population, with higher selection coefficients leading to more rapid purging and lower selection coefficients leading to patterns of interference among deleterious alleles. The recombination rate between a deleterious and a neutral allele will influence the number of generations they remain in association. All of these previous factors are present in classical derivations of the effects of background selection in single populations. In addition, when tracking the local effects of background selection, we add the expectation that the migration rate will influence the association of a neutral allele and deleterious allele *within the focal subpopulation*, as the emigration of a deleterious haplotype from the subpopulation will end the local effect of the deleterious allele on the reproductive success of the neutral allele.

Following Ref. [32], we consider two cases corresponding to whether a neutral allele is in coupling (case 1) or repulsion (case 2) with a deleterious allele in a single diploid individual. In case (1), the local association between the neutral and deleterious allele only persists if the haplotype isn't removed by selection, does not recombine, and doesn't leave the population

through migration, which happens with probability $(1-t)(1-m)(1-r) = (1-m)(1-t-r')$. Thus, the fraction of descendants of the neutral allele in the same subpopulation that carry deleterious allele i, τ generations later, is $[(1-m)(1-t_i-r_i')]^{\tau}$. Next, we must add the cumulative effects over time on the frequency of the neutral allele due to its association with deleterious allele i, also considering that we expect the deleterious allele to decrease in representation by t_i. Taking the sum from $\tau = 0$ to ∞,

$$\delta_{c,i} = \sum_{\tau=0}^{\infty}(t_i[(1-m)(1-t_i-r_i')]^{\tau}),$$

the expected net reduction in frequency of a neutral allele that is initially in association with deleterious allele i in this subpopulation over all generations is

$$\delta_{c,i} = \frac{t_i}{m+r_i'+t_i-mr_i'-mt_i}.$$

In case (2), the deleterious allele has an average reduction in frequency of t_i in the initial generation because of the presence in the same individual of the deleterious allele in repulsion, which only affects the local drift if that individual has not migrated. During this generation, there is a r' probability that the deleterious allele recombines onto the background of the neutral variant. If it does so, then the future reduction in fitness while it is in the same local population is the same as in case 1, except we modify case 1 by adding a factor r'_i, the probability that the neutral allele recombines onto the background of deleterious allele i . Therefore,

$$\delta_{r,i} = t_i\,(1-m)\; + r'_i \sum_{\tau=0}^{\infty}(t_i[(1-m)(1-t_i-r_i')]^{\tau}).$$

Taking the sum from $\tau = 0$ to ∞, we find that the expected net reduction in neutral allele frequency here is

$$\delta_{r,i} = t_i(1-m+\frac{r_i'}{m+r_i'+t_i-mr_i'-mt_i}).$$

These $\delta's$ may be regarded as a measure of the total reduction in fitness of a neutral allele due to its cross-generational association with deleterious allele *i*. Given that we expect a total fraction q_i of deleterious allele at locus *i* , we expect that the total additive genetic variance at the neutral site over time, due to its associations with the deleterious allele through Case (1) and (2), increases the variance in reproductive success by $\Delta V_i \approx q_i(\delta_{c,i} + \delta_{r,i})$ at the neutral site [C 2012]. Given the well-known result that $\frac{N_e}{N} = \frac{1}{1+\Delta V_i}$ [50], we may now use this to estimate $N_{e,local}$ at the neutral site experiencing background selection. Extending this to multiple deleterious sites in association with the neutral site at the local population, $\frac{N_{e,local}}{N_{local}} = \prod_i \left(\frac{1}{1+\Delta V_i}\right) \approx \prod_i (1 - \Delta V_i)$ for small ΔV_i. Using $\Delta V_i \approx q(\delta_a + \delta_r)$, we find that

$$\frac{N_{e,local}}{N_{local}} = B_{local} = \exp[-\sum_i \mu_i t_i (\frac{1+(1-m+r(1-t)-(1-t)(1-r)(1-m)^2)^2}{(m_i+(1-m_i)(r_i+t_i-r_i t_i))^2})], \qquad \text{(ST1)}$$

which for small values of r_i and t_i is approximately

$$B_{local} \approx \exp[-\sum_i \frac{\mu_i t_i}{(m_i+(1-m_i)(r_i+t_i-r_i t_i))^2}]. \qquad \text{(ST2)}$$

For a chromosome with total deleterious mutation rate *U* and map length *M* (in Morgans), the methodology used by Hudson and Kaplan [2] can be used to find that the above formula is well approximated by $B_{local} \approx \exp[-\frac{2Ut}{(t+m)(M + 2t + 2m)}]$. This requires a few assumptions: the neutral site is embedded in the center of a large region subject to deleterious mutations, *t* and *m* are fixed, and r_i is sufficiently small such that it is additive over sites. When $m = 0$, this formula reduces to $B_{local} \approx \exp\left[-\frac{2U}{2t+M}\right]$ [2].

For parameters such that we are in the *interference selection regime*, we then utilize the methodology of Good et al. [20] in order to output parameters suitable for our derivation, thus allowing us to make predictions when interference is common. Below, we discuss the application

of our derivation to the framework of Good et al. [20]. Then, we discuss the accuracy of these predictions under various parameters.

Prediction of B_{local} using the Good et al. [23] method

To derive our theoretical estimates of background selection in the *interference selection regime* in structured populations, we calculated *B* using the Python scripts supplied by Good et al. [23]. Briefly, Good et al. [23] found that the effects of background selection on neutral genetic diversity over an effectively non-recombining region, when interference is common, is determined by variance in fitness. They developed a method that approximates the effects of many weakly deleterious mutations in the *interference selection regime* as if there are fewer, strongly deleterious mutations in the *background selection regime* that generate equal variance in fitness. *B* is then estimated using these "effective parameters" in the *background selection regime* following classic background selection theory (i.e. Ref. [1, 3–4]), although the formula used by Good et al. [23] slightly differs from that of previous formulae as they include higher order corrections. The formula used is:

$$B \approx e^{-\lambda} + \frac{2(\lambda)e^{-\lambda}}{Nt}\int_0^1 z\log\left(\frac{1}{1-z}\right)e^{\lambda z^2}dz \qquad \text{(ST3)}$$

(Ref. [23] Eq. 2). Here, *t* is the selection coefficient against a deleterious allele in haploids (equivalent to the heterozygous selection coefficient in the case of diploids), *N* is the number of chromosome copies (i.e., population size for haploids and twice that for diploids), and

$$\lambda = \frac{2U}{2t+M} \qquad \text{(ST4)}$$

(described by Good et al.[23] as λ_{eff}), where *M* is the total recombination rate in a selected region in which a neutral site is embedded in the center and *U* is the gametic deleterious

mutation rate of the selected region. The parameters *U*, *t*, and *M* represent the "effective parameters" following use of the Good et al. [23] method.

To predict B_{local} using our derivation including the effect of migration, we opt to use Eq. ST3 of Good et al. [23], where instead

$$\lambda = \frac{2Ut}{(t+m)(M+2t+2m)}\,. \qquad \text{(ST5)}$$

Therefore, we account for higher order corrections, migration, and selective interference by employing Eq. ST3 with the rate parameter described by Eq. ST5 to predict B_{local} under varying *U*, *t*, *M*, and *m*. Using B_{local}, we then employ *Theory* Eq. 4 from the main text to get $F_{ST,\, predicted}$.

We used the following cut-off for determining whether a particular parameter combination corresponded to the *interference selection regime*: $N_{local}te^{-\frac{2U}{2t+M}} < 2.5$ [23]. The precise formalization of the boundary between the *interference selection* and *background selection regimes* remains an unsolved problem in the background selection literature, but we found the aforementioned cut-off to be reasonable.

Evaluation of Good et al. [23] method in predicting F_{ST} and π_T

Below, we present predictions from our application of the Good et al. [23] method with and without migration to our simulations of F_{ST} under low and high migration, and with some and no recombination (Fig A). Here, we can see that the Good et al. [23] theory in conjunction with the 'migration effect' derivation accurately predicts F_{ST} across the data sets (Fig A) as well as quantitative genetic models of background selection (see main text Fig 1). One caveat, however, is the lack of accuracy for estimates at $N_{local}t = 2.5$ under our low migration parameter set. This is to be expected, as this corresponds to the chosen boundary between the *interference* and *background selection regimes* in our prediction of B_{local}. Moreover, as expected, we see that,

in the absence of the ‘migration effect’, the Good et al. [23] method erroneously predicts an inverted U-shaped curve of F_{ST} with respect to $N_{local}t$ under our high migration simulations (Fig A.A & A.B), and is less accurate in predicting F_{ST} overall.

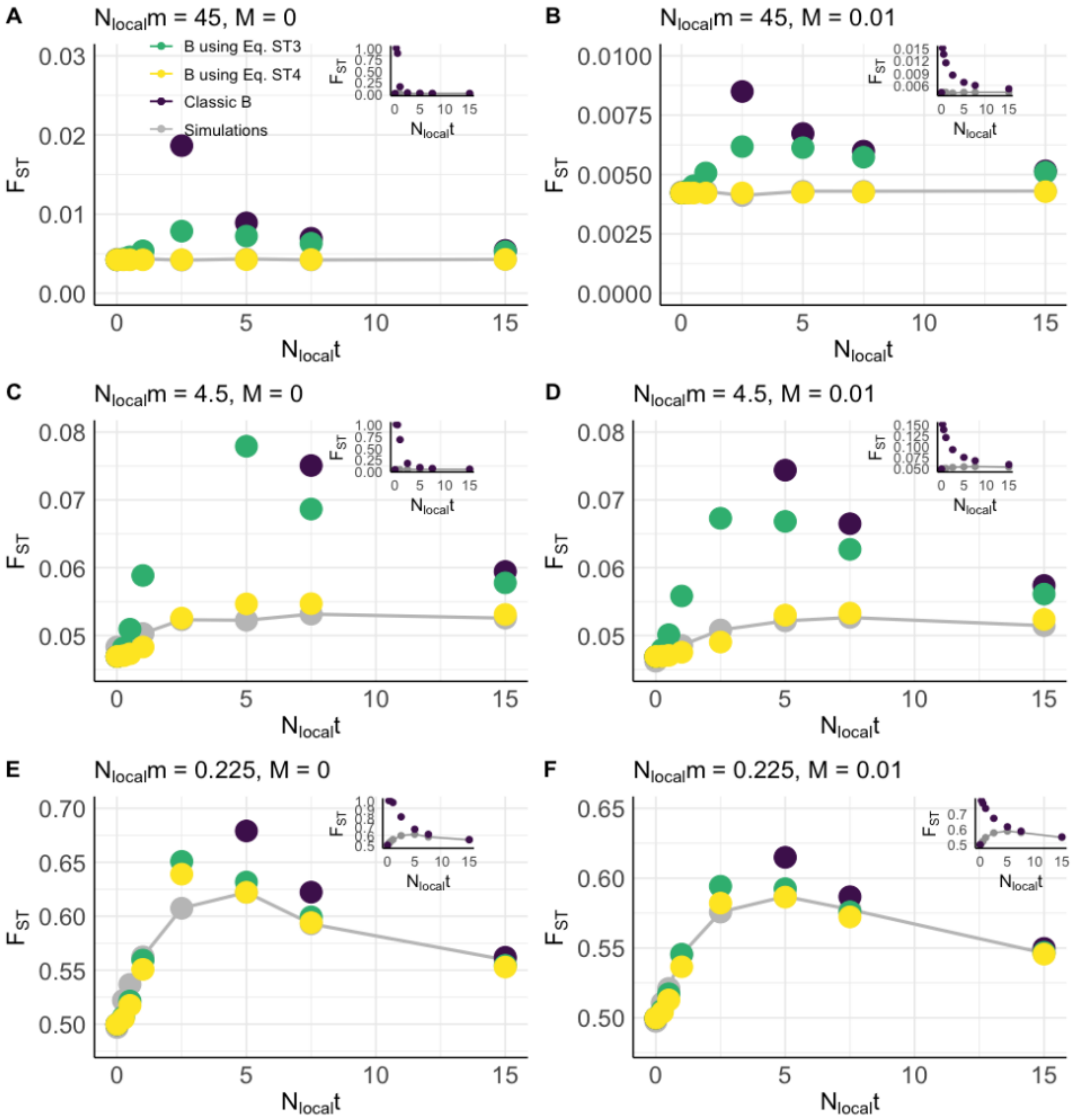

Fig A. Accuracy of various methods in predicting F_{ST} with respect to the effective heterozygous strength of selection, $N_{local}t$. Forward-time simulations run with (A)

$N_{local}m$ = 45 and M = 0, (B) $N_{local}m$ = 45 and M = 0.01, (C) $N_{local}m$ = 4.5 and M = 0, (D) $N_{local}m$ = 4.5 and M = 0, (E) $N_{local}m$ = 0.225 and M = 0, and (F) $N_{local}m$ = 0.225 and M = 0.01. The gray dots connected by a line represent F_{ST} values from forward-time simulations, with 95% CIs. (These were quite small, and not always visible.) The yellow dots represent numerical predictions from our application of the Good et al. [23] method to structured populations with consideration of the 'migration effect' to predict B_{local} (Eq. ST3 & ST5), and the green dots without its consideration (Eq. ST3 & ST4). The insets show the performance of classic background selection theory (purple dots; *Methods;* Eq. 7) to structured populations following Ref. [36]. All parameter combinations were simulated 750 times with local population size N_{local} = 500, scaled selection coefficients $N_{local}t$ = {0, 0.25, 0.5, 1, 2.5, 5, 7.5, 15}, U = 7x10^{-3}, $L_{selected}$ = 700, and for 250,000 generations. In many cases (especially in part D), the plot points for the simulations (gray dots) are hidden behind the points shown for theoretical expectations.

We also test the Good et al. [23] method with the migration effect over various other migration rates, finding that it too accurately predicts F_{ST} under various parameters (Fig B). Note that the incorporation of the migration effect results in more accurate predictions of F_{ST} than without (*cf.* Fig B yellow dots to green dots)

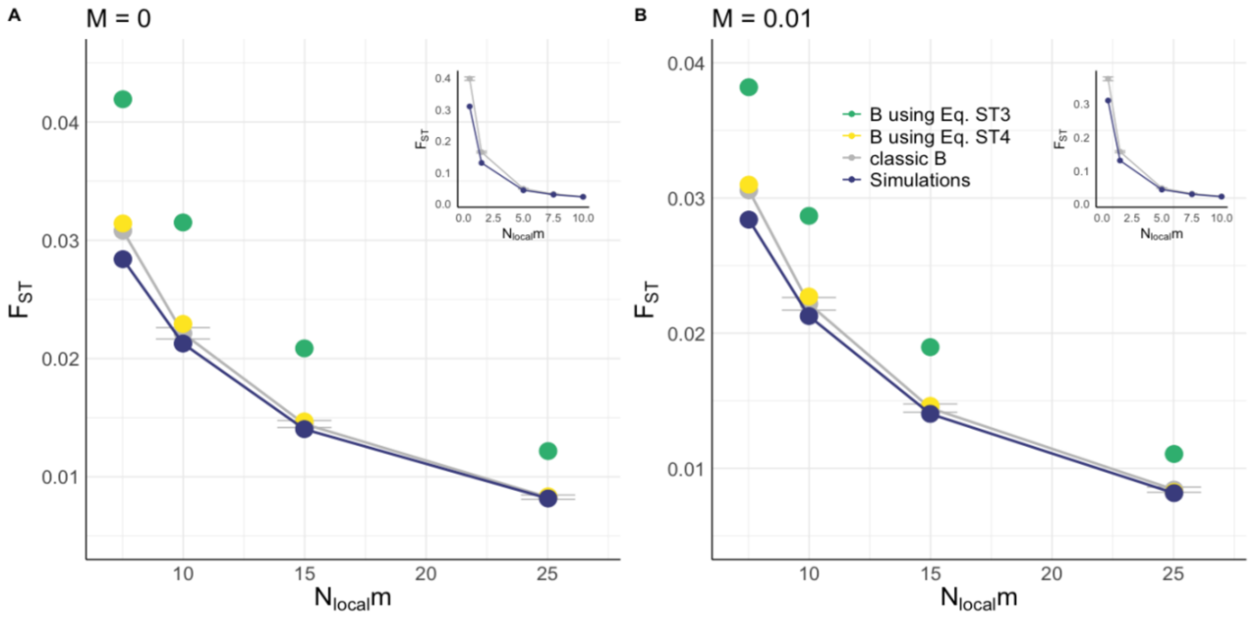

Fig B. Inclusion and exclusion of migration effect in estimation of F_{ST} under background selection. Forward-time simulations under varying rates of migration for (A) $M = 0$ and (B) $M = 0.01$. The gray dots represent observed F_{ST} and are connected by a line. The yellow dots represent our application of Good et al. [23] with the 'migration effect' to predict B_{local} (Eq. ST3 & 5), the green dots represent the Good et al. [23] method without the 'migration effect' (Eq. ST3 & ST4), and the dark blue dots are F_{ST} from simulations run without deleterious alleles (F_{ST} under mutation-drift balance). The insets zoom in on cases for which $N_{local}m \geq 7.5$. All simulations were run 750 times in a 10-deme island model with $N_{local} = 500$, $U = 7\text{x}10^{-3}$, $L_{selected} = 700$, $N_{local}t = 7.5$, and for $50N_{global}$ generations.

Below, we also show the Good et al. [23] predictions of π_T and π_S in a metapopulation. Similarly to predictions from quantitative background selection theory presented in the main text, we find that the Good et al. [23] method inaccurately predicts π_T and π_S under low effective

strengths of selection (Fig C & D $N_{global}t \leq 25$), with this discrepancy being more substantial for our low migration parameter set.

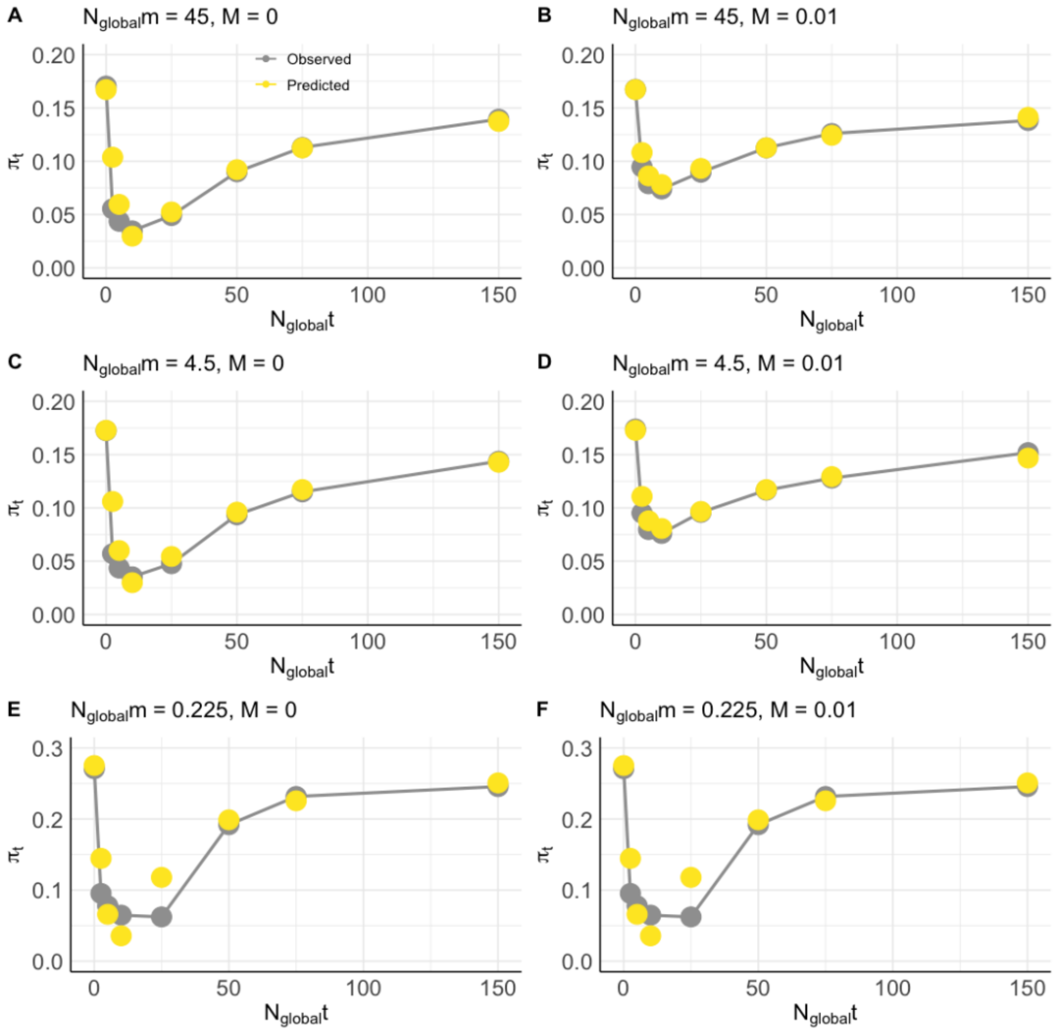

Fig C. Predicted π_T versus observed π_T in a metapopulation. Forward-time simulations run with (A) $N_{local}m$ = 45 and M = 0, (B) $N_{local}m$ = 45 and M = 0.01, (C) $N_{local}m$ = 4.5 and M = 0, (D) $N_{local}m$ = 4.5 and M = 0, (E) $N_{local}m$ = 0.225 and M = 0, and (F) $N_{local}m$ = 0.225 and M = 0.01.. The gray dots represent π_T from forward-time simulations using a 10-deme island model with N_{global} = 5000, $N_{global}t$ = {0, 2.5, 5, 10, 25, 50, 75, 150}, m = {0.00045, 0.09}, R = *{0, 0.01}*, and are connected by a line. The yellow

dots represent theoretical predictions of subpopulation diversity using B_{local} (Eq. ST3 & 5; see *Methods Calculation and Prediction of* π_T) with incorporation of the migration effect to estimate G_{ST}, (*Theory* Eq. 5), $N_{e,\ global}$, and therefore π_T.

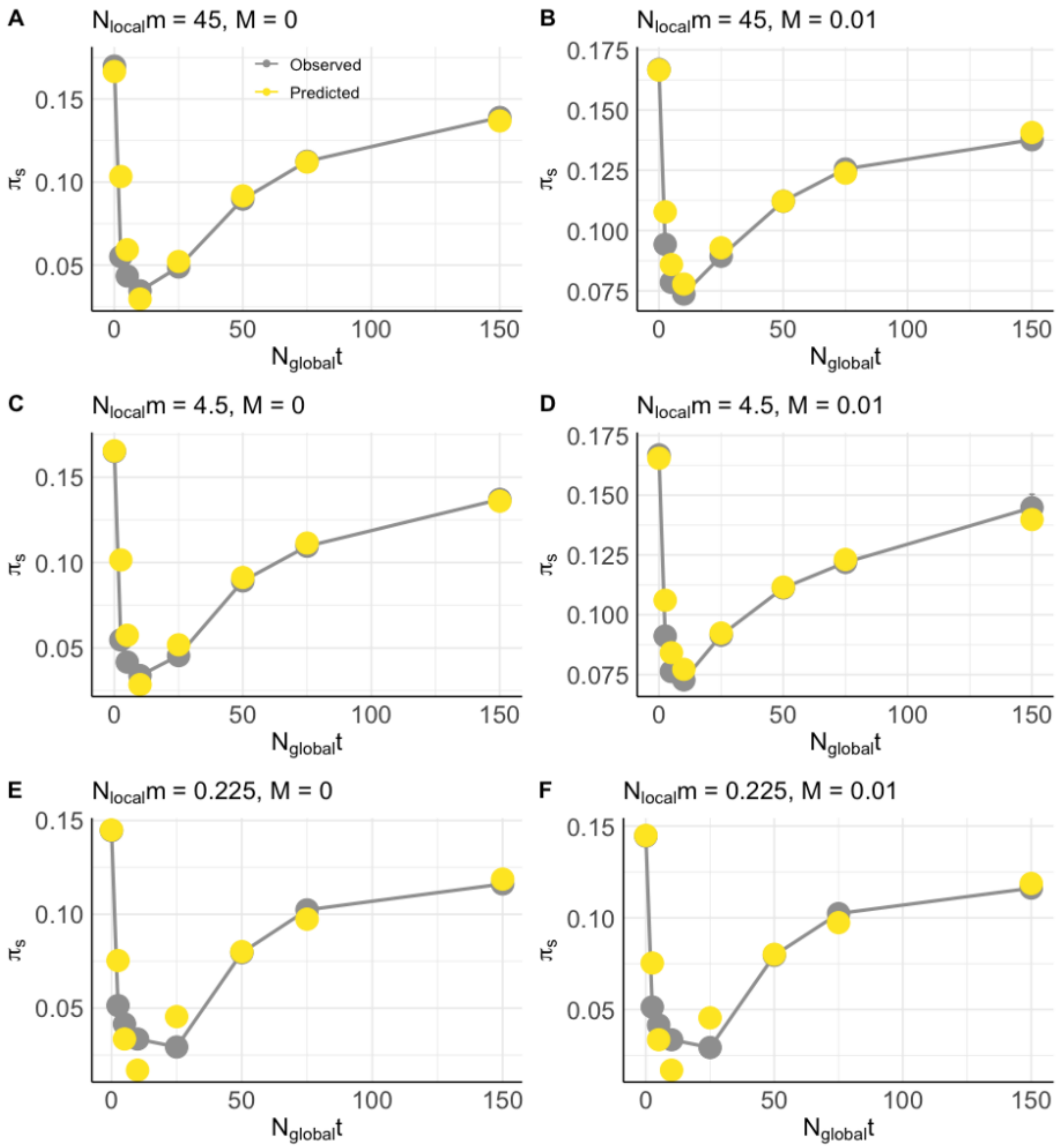

Fig D. Predicted π_S versus observed π_S in a metapopulation. (A) Forward-time simulations run with $N_{local}m$ = 45 and M = 0, (B) $N_{local}m$ = 45 and M = 0.01, (C) $N_{local}m$ = 4.5 and M = 0, (D) $N_{local}m$ = 4.5 and M = 0, (E) $N_{local}m$ = 0.225 and M = 0, and (F)

$N_{local}m$ = 0.225 and M = 0.01.. The gray dots represent forward-time simulations using a 10-deme island model with N_{global} = 5000, $N_{global}t$ = {0, 2.5, 5, 10, 25, 50, 75, 150}, m = {0.00045, 0.09}, M = {0, 0.1}, and the yellow dots represent theoretical predictions of total diversity using the Good et al. [23] method (Eq. 3; see *Methods Calculation and Prediction of* π_T) with incorporation of the migration effect.

The modified code of Good et al. [23] used to generate predictions of B_{local} with consideration of the effects of migration may be found at https://github.com/asadrh8/FST-BGS.
