# Supplementary figures and images for: FST and genetic diversity in an island model with background selection
Source: PLoS Genet. 2024 Dec 2;20(12):e1011225. doi: 10.1371/journal.pgen.1011225 (PMC11637402; doi:10.1371/journal.pgen.1011225)

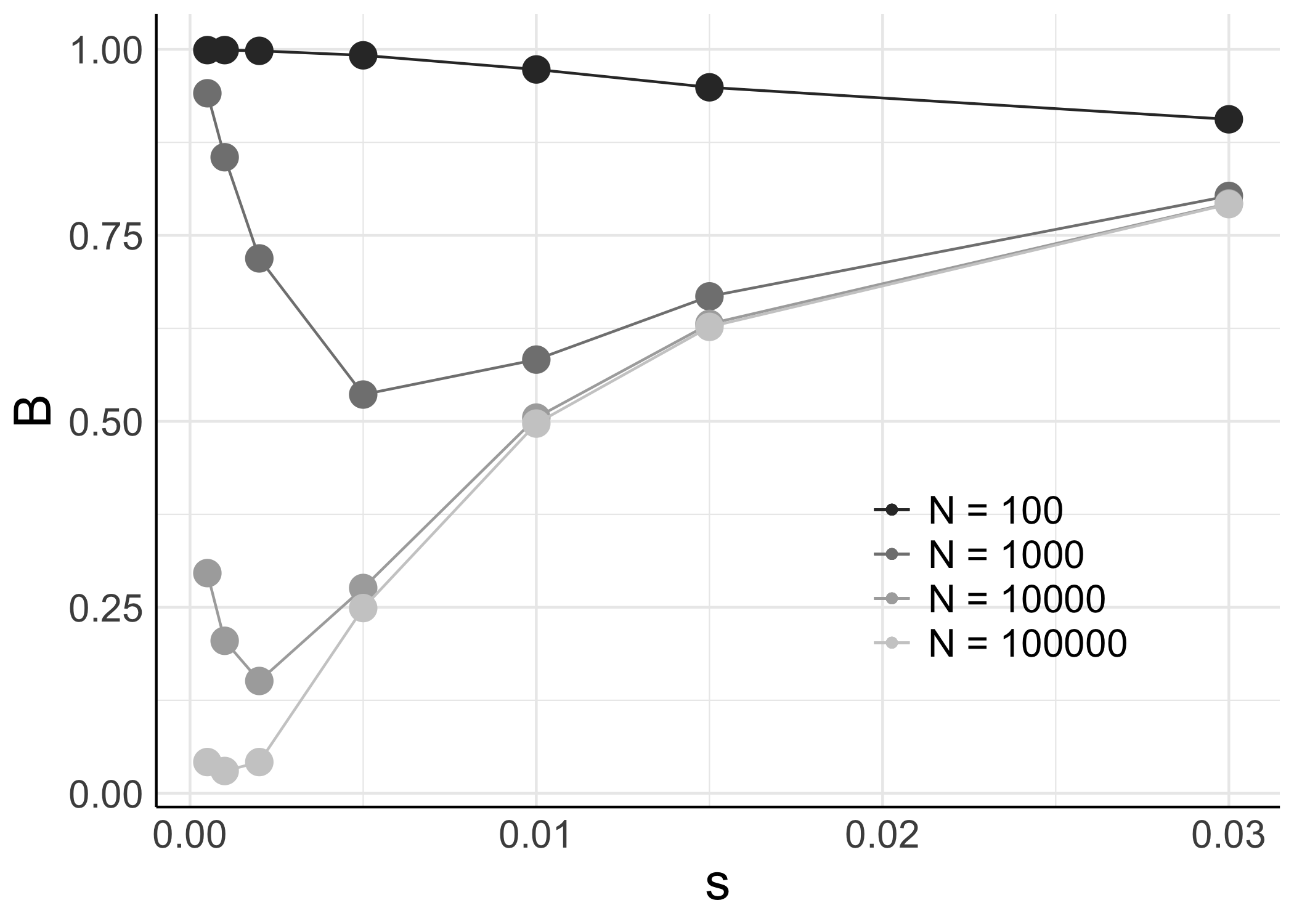

Supplement: S1 Fig — B was computed for haploid deleterious selection coefficients of s = {0.0005, 0.001, 0.002, 0.005, 0.01, 0.015, 0.03}, haploid population sizes of N = {100, 1000, 10000, 100000}, U = 7x10^-3^, and M = 0. Here we see that, as N increases, the theory predicts greater reductions in B for weaker selection coefficients (s ≤ 0.005). (TIFF) [file pgen.1011225.s003.tiff]

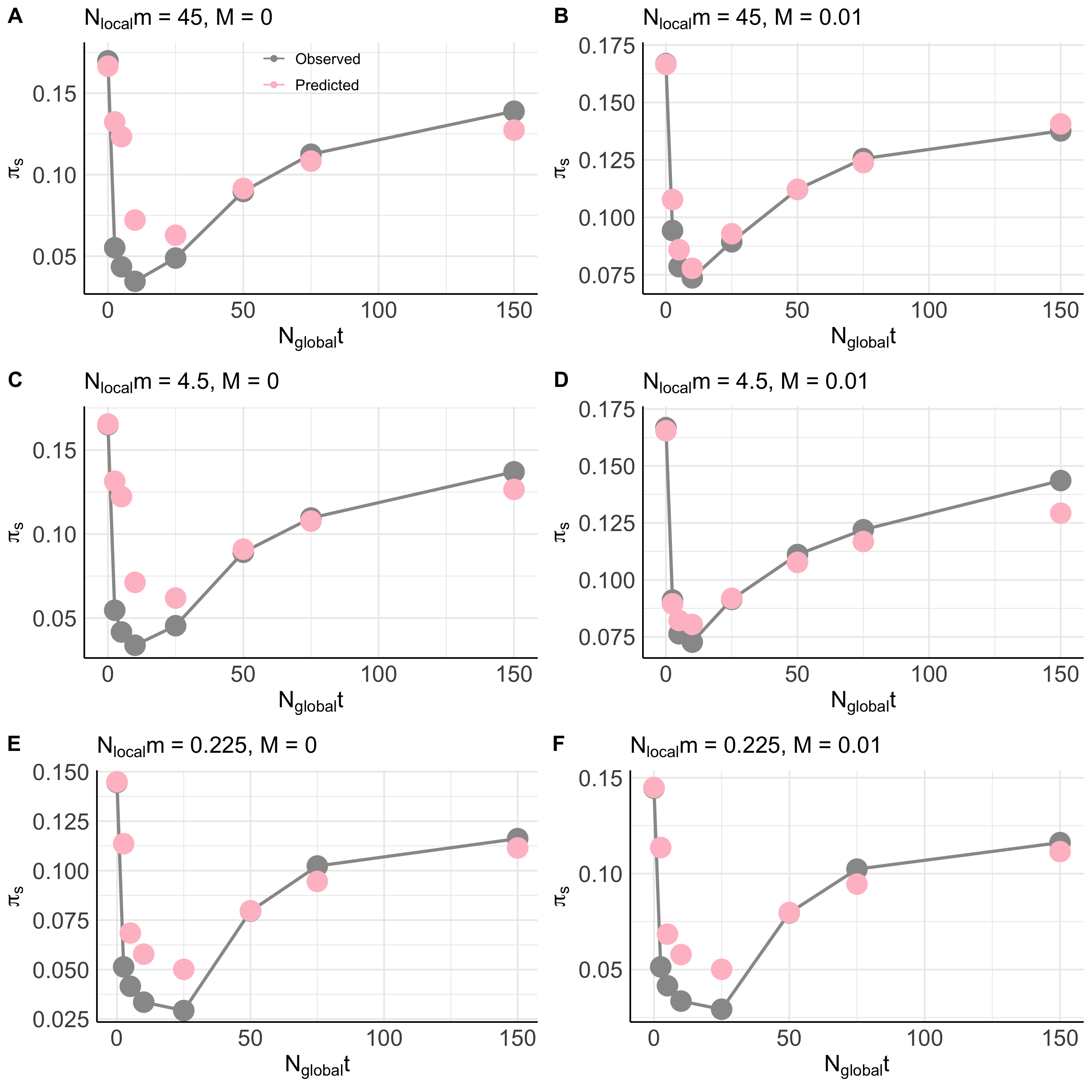

Supplement: S2 Fig — Forward-time simulations run with (A) Nlocalm = 45 and M = 0, (B) Nlocalm = 45 and M = 0.01, (C) Nlocalm = 4.5 and M = 0, (D) Nlocalm = 4.5 and M = 0, (E) Nlocalm = 0.225 and M = 0, and (F) Nlocalm = 0.225 and M = 0.01. The gray dots represent πS from forward-time simulations using a 10-deme island model with Nlocal = 500, Nlocalt = {0, 0.25, 0.5, 1, 2.5, 5, 7.5, 15}, m = {5x10^-5^, 1x10^-2^}, and are connected by a line. The pink dots represent theoretical predictions of total diversity (see Methods) using predictions of Blocal with incorporation of the migration effect (Eq 2) to estimate GST, (Eq 5), Ne, global, and therefore πS. (TIFF) [file pgen.1011225.s004.tiff]

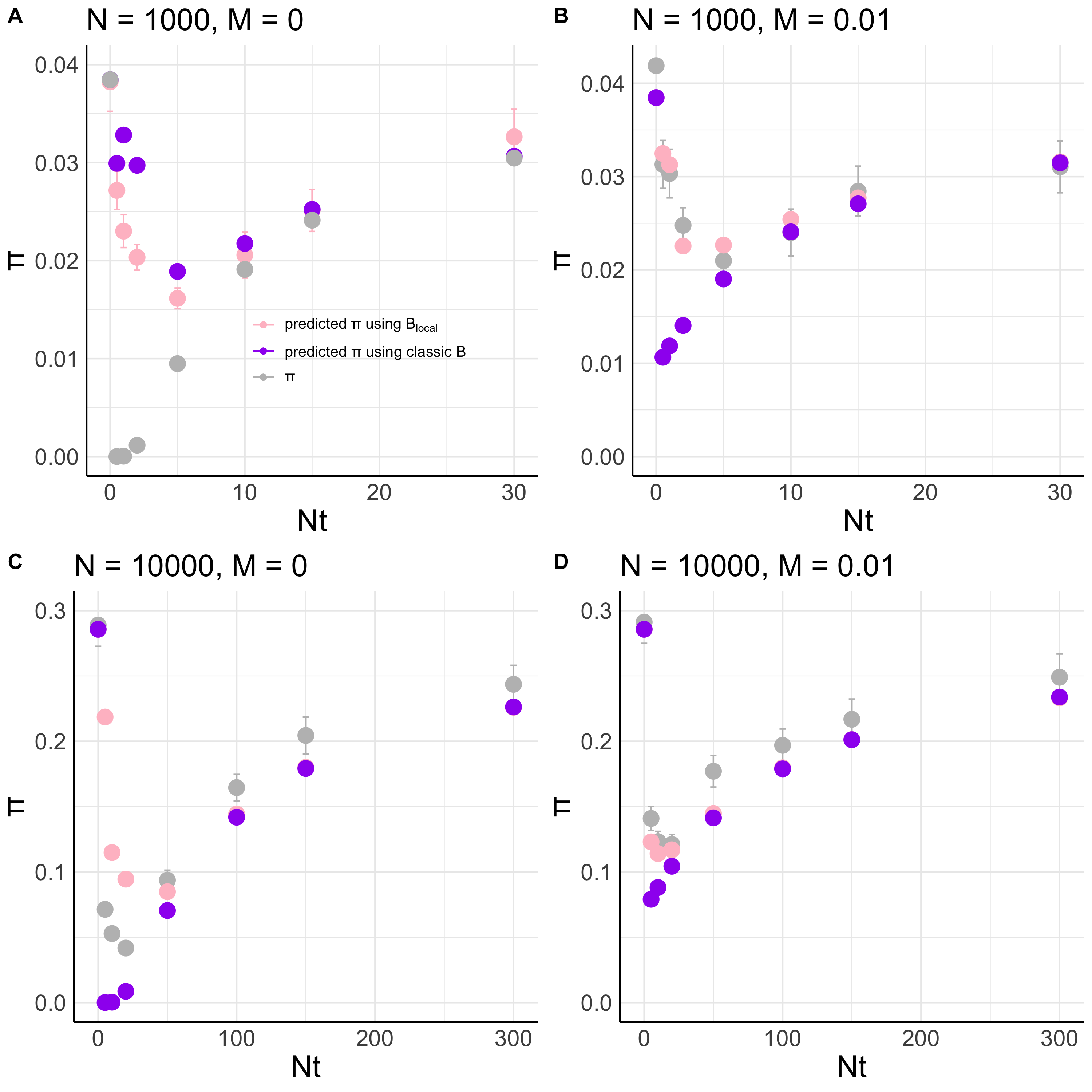

Supplement: S3 Fig — Forward-time simulations of a panmictic population run with (A) N = 1000 and M = 0, (B) N = 1000 and M = 0.01, (C) N = 10 000 and M = 0, (D) N = 10 000 and M = 0.01, The gray dots represent observed genetic diversity, π, from our panmictic simulations. The pink dots represent predicted π using the model of Buffalo & Kern (2024) (Eq 2), equivalent to our model when m = 0, and the purple dots represent predicted π using classic B (Eq 7). (TIFF) [file pgen.1011225.s005.tiff]
