# Supplementary material for: FST and genetic diversity in an island model with background selection
Source: PLoS Genet. 2024 Dec 2;20(12):e1011225. doi: 10.1371/journal.pgen.1011225 (PMC11637402; doi:10.1371/journal.pgen.1011225)
Supplement: Attachment — Submitted filename: Hasan-Whitlock-comments.pdf [file pgen.1011225.s006.pdf]

General comments

The strength of this work is that it provides an improved theoretical model of the effect of background selection in an island model of a subdivided population, with better insights into its effect on diversity and population differentiation as measured by F_{ST}. In particular, they give a treatment of the interference selection regime, based on work by Good et al. (2014). The validity of the theory is tested against simulations. Since natural populations are often subdivided, such a theoretical advance is welcome.

The weakness relates to the realism of the assumptions used in the simulations and illustrations of the theoretical results. In the Methods (l.548-9), they say rather vaguely that their parameters are "biologically realistic on average", but acknowledge that they were not trying to simulate real organisms. But how realistic are their parameters? They use a genome model of 10^4 neutral sites surrounded by two blocks of 350 selected sites. They don't make it clear what this is supposed to represent; are these two small exons abutting a large intron, or a large intergenic region between two small exons? It certainly doesn't represent even a single gene, and of course a lot of published data come from synonymous sites which are embedded in numerous selected sites.

Their total population size is 5000, and their non-zero recombination rate is r = 1.41×10^{-5}. It's a good rule for simulations to ensure that the deterministic parameters scaled by effective population size are consistent with what you expect in a much larger natural population. They don't explicitly mention this, nor do they say what type of organism they have in mind. Let's assume that the total N_e is like that of a medium-sized mammal, 10^5 say. Then their Nr = 0.057 would correspond to $r = 5.7 \times 10^{-7}$; this is very high compared with a typical r of 10^{-8}. Even an N of 10^6 gives an r that looks high. Similarly, their deleterious mutation $U = 7 \times 10^{-3}$ gives a per basepair mutation rate of 1×10^{-5}; this corresponds to a mutation rate of 5×10^{-7} for $N = 10^5$ and 5×10^{-8} for $N = 10^6$, both rather high when compared with published estimates. Similarly, it's not clear how their choice of selection coefficients correspond to the estimates obtained from population genomic studies, which suggest that the mean selection coefficient scaled by N_e is of the order of 10^3 or more, with a wide distribution around it. In the case of an island model, Wakeley (2003 Genetics 163:411) showed that the scaled selection parameters that control the distribution of allele frequencies correspond approximately to the product of selection coefficient and N_e for the whole population, total $N/(1–F_{ST})$, if subdivision is not too extreme. An $N_{local}t$ of 15 would thus correspond to an N_et of 15 with a neutral F_{ST} of 0.5 and total N=10^5. Their strongest selection case is thus actually rather weak compared with the typical range of values suggested by the population genomics data.

A weakness of most of the theoretical analyses of the interference selection limit is that they ignore reverse mutations, so they don't realistically predict the stochastic equilibrium situation that is likely to be reached with base substitutions; an exception is Devi et al (2023 Genetics 224: iyad091).

Finally, they don't say what they assume about the neutral mutation rate used to generate their diversity values. Looking at their Fig.3, these seem extremely high compared with most observed values for silent sites in natural populations. If they assumed the same mutation rate as for selected sites, 1×10^{-5}, $4Nu$ would be 0.2, which presumably explains these high values, for which the usual infinite sites assumption used in the relating BGS predictions is somewhat inaccurate.

Overall, therefore, it's not clear that their results give a good guide as to what you might expect to see in population genomic data on real organisms. They strongly emphasise the importance of the interference selection limit, but the available data on the distributions of selection coefficients for deleterious mutations suggest that only a small fraction of mutations fall into this class (e.g., Kim, B et al 2017 Genetics 206:345). They may be important for the properties of low recombination rates of the genome, where N_e is strongly reduced by interference, but it's unlikely that this is true in many parts of most genomes. The statement on l.454-56, that "most deleterious alleles are typically expected to be weakly deleterious and therefore suited to the interference selection regime" is thus misleading. In addition, in regions of the genome where recombination is low and interference is high, associative overdominance effects may also be operating, confounding some of the expectations under pure BGS; this possibility is not mentioned (Becher et al 2020 Curr Biol 30:94; Gilbert et al. 2020 Curr Biol 20:101).

Specific comments

l.60 Qualify by saying that they have to be sufficiently strongly selected.

l.72 The new paper by Buffalo & Kern should be cited in a revision (2024, PloS Genet 20:e1011144). This uses an approach to predicting *B* with weak selection based on Santiago & Caballero 2016 (Genetics 204: 1267), which is less impenetrable than that of Good et al.

l.73 Recent work on this by Parul Johri and others should be cited.

l.78 It would be more accurate to say that the modelling of BGS involves pairwise coalescent times; this accurately predictsthe π values under the infinite sites assumption (not met accurately in the present simulations).

l.86 The 1st application of this idea to BGS was in ref. 3.

l.96 This is only one definition of F_{ST}; others are frequently used in relation to population genomics data, as is mentioned much later.

l.103 It's odd not to mention ref. 32 here, which drew attention to this explanation for high F_{ST} in low recombination regions of Drosophila.

l.116 Again, the effect of weak selection was recognized much earlier than ref. 29 (see ref. 34).

l.138 There's a follow-up paper (Melissa et al. 2022 Genetics 221:iyac053), which modifies the conclusions somewhat (I don't know how important this is).

l.159 Ref. 7 is very old for this topic; see my general comments.

l.180 What about low recombination regions, which can be quite large in many organisms? There's increasing evidence that F_{ST} is elevated in such regions, e.g. Burri et al. 2015 Genome Res. 25**:** 1656.

l.253 The justification for this relation should be mentioned.

l.285-286 Is this really very important- if all forces are weak, it really shouldn't matter. If migration is strong, then subdivision is not important.

l.325 This is presumably a reflection of the same effect observed for diversity, e.g. by Gordo et al. 2002 Genetics 78: 835.

l.345 The usual term is 'scaled selection coefficients'.

l.352 This effect was already mentioned in ref. 1.
