# Supplementary material for: FST and genetic diversity in an island model with background selection
Source: PLoS Genet. 2024 Dec 2;20(12):e1011225. doi: 10.1371/journal.pgen.1011225 (PMC11637402; doi:10.1371/journal.pgen.1011225)
Supplement: Attachment — Submitted filename: response_to_reviewers.pdf [file pgen.1011225.s007.pdf]

Dear Dr. Payseur and Dr. Fay,

Thanks for your and the reviewers' comments on our paper. They are very insightful and have been very helpful in making a revision, which we here submit.

Throughout this response letter, we will share the comments from you or the reviewers in *black*, and our responses will be in blue Roman face.

You will see that all reviewers commend the manuscript for reporting a solid theoretical advance. They (and I) agree that extending predictions under background selection to subdivided populations is an important goal. In contrast, two of the reviewers are skeptical about the broader significance of the work. This skepticism, which I share, stems primarily from three issues:

We are glad that the reviewers like aspects of the manuscript. We will add general responses to the three main points below, and then expand on these in the comments from the individual reviewers.

(1) the manuscript reports no comparison of model predictions to empirical data,

We believe that we were not clear in the original version about our intentions for the paper. We did in the first version (and indeed still) talk generally about the kinds of expectations that the theory predicts for natural populations. But our main intent is to (1) ask whether the many approximations involved in previous derivations of the effects of BGS on F_{ST} are sufficiently accurate, and (2) to derive new theory that improves on the accuracy of the approximations. In order to test the approximations involved in a theoretical treatment, it is necessary to do simulations where we both know the true demographic and genetic parameters that make up the input of the theory and can measure the effects on response variables without confounding variables that do not factor into the theory itself. So our simulations are intended to ask whether the theory works, in the sense of "under the conditions described by the theory, "Does the theory make an accurate prediction?".

We find that previous theory doesn't predict F_{ST} well under all circumstances and develop new theory that we show does work well. Drawing comparisons to empirical data actually doesn't help us know if the theory is accurate, because we do not know the true values of the demographic parameters (e.g. population sizes, migration rates) that are necessary to make predictions with the theory.

Applying this to empirical data is a non-trivial task which has taken others in previous works substantial effort (e.g. McVicker et al.), which does not help us understand the accuracy of the theory better. As such, it is not a focus of the paper.

(2) assumed parameter values may not be realistic,

We have four main responses to this. First, we think we described our parameter choice a little too cavalierly – we think that all of the parameters we used were biologically reasonable. We have edited the paragraph describing the parameter choice and added a section at the beginning of the simulation section which describes the relationship of the parameter choice to the goals of the paper more directly.

Second, please see our responses to Reviewer 3, where we spell out several better justifications (now enhanced in the paper) for our parameter choices and show how they relate nicely to realistic values. (Some of our wording in the original draft was insufficient for the reviewer to see what we had done, given the oddities of SLiM.)

Third, we have added calculations and simulations on an intermediate level of gene flow, corresponding to approximately $F_{ST} = 0.05$.

Finally, given that the intent of the paper is to test the effects of assumptions in the derivations of the theory, some of our choices are guided by what will make good tests. For example, we made sure that we covered cases where we expected the theory to be most inaccurate. Still, to do those tests effectively, it is important that the parameters chosen are in a biologically reasonable range, which we believe is the case.

and (3) the effects of background selection tend to be weak (especially on Fst).

The effects of background selection on F_{ST} ARE weak, but that is one of the main points of the paper. We show here that previous analyses (i.e. Zeng and Corcoran) greatly overestimate the consequence of BGS on F_{ST} in cases with non-trivial migration. We think it is relatively important to correct this in the literature.

To be reconsidered for publication, these concerns would need to be answered convincingly. I would understand if you decide to submit to a journal where connections to real data are less salient than PLoS Genetics.

We believe that the paper as it stands reports on a significant theoretical advance, and we cannot find anything in the instructions to authors of the journal that indicates a need for empirical data. Of course, you better understand than we what the scope of the journal is. If you do not think the paper as it stands is suitable in terms of its subject matter, we would be grateful if you could please return it to us soon before review.

Thanks again for your useful comments and for your efforts for this review process.

Sincerely,
Asad Hasan and Mike Whitlock

Reviewer #1: In this work, Hasan and Whitlock derive theory for the reduction in effective population size due to background selection (BGS) in a metapopulation model under both strong and weak BGS. The authors do this by finding the appropriate rate parameter for the Good et al. (2014) model under migration (their equation 8). Using forward simulations, they show their work has a close correspondence with their theory.

While I think this is nice theoretical work and helps us refine our expectations of the impact of BGS in more complex situations with migration, the paper would be much stronger with at least one empirical application.

Major Comments

- Throughout the paper, the authors frame a lot of the discussion of BGS in terms of fitness variance. Recent work in quantitative genetic approaches to BGS (Santiago and Caballero, 2016 and Buffalo and Kern, 2024) frame things similarly and provides theory to predict reductions in diversity in terms of fitness variance. These models are arguably easier to build intuition around than the Good et al. (2014) method. Since this paper is trying to incorporate weak selection BGS theory with migration, it would be strengthened by trying to frame their theory in terms of these more intuitive models that seem to work quite well for Eukaryotic genomes (though they have remaining issues when 2Ns ~ 1). Is there a way to frame equations 2-3 in terms of the Santiago and Caballero model? I would also consider citing this recent work on weak BGS since it is quite pertinent to the present study.

This is an excellent point. We have now redone the analysis from the Santiago and Caballero/ Buffalo and Kern approach, and reach the same conclusions. It is nice to have all of the work in the same framework, as the reviewer suggests.

- As mentioned, this paper would be greatly strengthened with some application to an empirical dataset. Does the new theory fit any data better than previous work? Are any conclusions drawn from previous work wrong under the new model?

Please see our response to the editor's first point.

- Line 86-87: There are many features (e.g. SFS) not captured by a simple rescaling of Ne under background selection models. I would include a sentence making this point, explaining that distortions in genealogies and thus the site frequency spectrum occur, and cite Good et al. (2014), Cvijović et al. (2018), and Good and Desai (2013). Cvijović et al. were cited earlier, but this is an important point to make to readers, that the BGS process is more than rescaling Ne in reality, even though that approximation works well.

Good point. We agree that this is an important aspect of background selection models that we had not previously made explicit. We have updated this paragraph, making the point that the rescaling of N_e is an approximation that does not capture all of the effects of background selection, but does reasonably well.

- Line 494: I found this section on Lewontin's Paradox speculative and inaccurate. While it is good to mention that the authors' theory leads to more accurate predictions of diversity and Fst under more complex demographic situations with migration, there is nothing in the present paper that indicates BGS will now lead to reductions in diversity over the several orders of magnitude necessary to explain Lewontin's Paradox. This issue of the scale of the problem was first made by Coop (2016), and later Buffalo (2021) quantified the relationship between π and Nc and explicitly tried to test whether BGS could explain the reductions, finding it could not. The reductions in the present paper, e.g. those in Figure 3 are at most ~ 0.05, but again, the observed shortfall in Buffalo (2021) is several orders of magnitude.

We agree that this paragraph is quite speculative and makes a claim that is unjustified by our work and in contrast to previous work on the topic. We have now removed this paragraph, and instead devote the majority of our discussion to discussing the effects of migration on how background selection influences π_S and π_T, and how our theory leads to more accurate predictions than previous work here.

Minor Comments

- Line 28: looks like a possible formatting error: π and T look like they are the same size.

Fixed

- Line 84: I would also cite Santiago and Caballero (2016) here, which extends this line of work, handling the weak selection case that Good et al's (2014) paper mentioned.

Agreed. We've add this reference here

- Lines 99-101: I would rework this paragraph's introduction, as two important points are being made: BGS affects structured populations and BGS is mediated by genomic organization. First, it is worth pointing out that it is not just recombination rate that mediates the effect of BGS across the genome, but also the placement of functionally conserved regions along the genome. I would add mention of this.

We agree. We have edited this paragraph to separate these points.

- Line 125: I would change the citation position here as U/t[1] could easily confuse readers as some mathematical notation.

Good point. Fixed.

- Line 138: I would also consider the work of Santiago and Caballero (2016) and Buffalo and Kern (2024) here. In these works, the weak selection issue is approximated using quantitative genetic models of BGS, with fitness variance rescaled according to the rate of fixation of weakly deleterious mutations.

This is an excellent point. We have now reworked the main results of the paper so that they are presented in terms of the logic of Santiago and Caballero (and Buffalo and Kern) more explicitly. We still keep the results based on Good et al. in an appendix (Text S1). We have added reference to these other papers here as well.

- Line 145: Rather than say "multiplying" I would say "rescaling".

Done.

- Line 212: Is this the first mention of Blocal? It needs to be described more clearly. What makes it local, e.g. is recombination or mutation deme specific?

The “local” here refers to the spatial location on the geographic landscape. We have now added an explicit definition in our derivation.

- Throughout, the authors should make sure "exp" is not formatted properly (not italicized). In LaTeX this would be "\exp".

Fixed

- I do not see any link to GitHub or another code repository website. It is imperative that all code, data, and analyses be accessible to reviewers and future researchers.

We agree. The code is now available on GitHub at https://github.com/asadrh8/FST-BGS.

Reviewer #2: In this manuscript, the authors derive expressions for the relative levels of neutral nucleotide diversity in the presence of background selection (BGS) while accounting for migration when selection is weak and the population is likely under the interference selection regime. They do so by extending the previously used approach presented in the Appendix of Charlesworth 2012 (which had accounted for selection and recombination but assumed panmixia) to account for migration and obtain the relative rate of coalescence in the classical BGS regime. Then they use Good et al.’s approach to account for interference and predict FST values as well diversity in the metapopulation.

Overall this is an important contribution to the field and I strongly recommend the acceptance of the submitted manuscript. I do, however, have a few suggestions involving writing as I believe that the manuscript can be improved to reach a broader audience.

Major comments:

-The authors have done a good job of indicating what work was done previously and what new work was performed in this study. However, that has resulted in the paper being somewhat scattered. It was hard for me to follow what exactly was done and how.

We have substantially re-written the theory sections. We think they are clearer now.

For instance, one reaches Figure 1 and it’s not clear at all what the different ways of calculating FST are. I would recommend clearly restating in the results section exactly how the green, yellow, and purple points were obtained.

We agree this was confusing. We have now simplified the figures, showing fewer theoretical cases. In each case we now point to specific equations in the text to highlight the intent of the legend.

-In your section “Derivation of B with ‘migration effect’ ”, it was easy to follow the derivation once I read through the Appendix of Charlesworth 2012. But it’s difficult to follow it independently. I would strongly recommend that the authors expand on the derivation a bit so that one does not have to read another paper to understand the derivation presented in the current study.

We have now added more details about the Charlesworth 2012 approach in this derivation (now found in S1 Text)

-Your equation on line 235 -> Can you please explain why your summation is not over [1 - t_i - r_i’ - m]^n , if we were to precisely follow the logic of the Appendix in Charlesworth 2012?

The equation in Charlesworth 2012 uses r’ = (1-t) r, so that the probability that neither recombination nor selection occurs is (1-t)(1-r) = (1-t-r’). In the present case, we need to find the probability that neither selection, recombination nor migration occur, which requires the form we give in the paper. We have added a comment to this effect.

-It would be highly useful if the authors presented the migration rate in terms of N_local*m, instead of simply “m” (this applies to all main figures). It’s very hard to understand the context of these migration rates. Relatedly, it would be helpful if the authors can discuss their results on high vs low migration rates in the context of N_deme*m – what are high and low migration rates? For instance, your high migration cases have N_local*m = 45, which is extremely high, such that the population would probably be considered a panmictic one in practice.

All of the relevant figures have been changed to be in terms of $N_{local}m$ instead of m. We agree that our high migration case is extremely high (representing a population that is almost panmictic). These parameters were chosen to illustrate that, despite the population being panmictic in practice, the action of migration itself may strongly influence the effect of background selection on F_{ST}, such that we observe no effect of background selection on F_{ST} due to high migration. That being said, we have modified some sentences after the first subheading of our Results section to make it clear to the reader that this is indeed on purpose.

-Please make publicly available the associated code and scripts used to generate the results in this paper.

Yes, please see above.

Minor comments:

-Line 455-456 -> You say that deleterious alleles are typically expected to be weakly deleterious. That is not what most studies have inferred. At selected sites, we usually observe a large proportion (at least 50%) of moderately and strongly deleterious mutations. The statement needs to be supported by citations or other reasons.

We agree that this statement is unsupported by current evidence. We have now reworded our statement to no longer make any claims about most deleterious alleles being weakly deleterious, and have provided an additional, more recent, citation (referencing the work of Kim et al. 2017) to our statement.

Reviewer 3:

General comments

The strength of this work is that it provides an improved theoretical model of the effect of background selection in an island model of a subdivided population, with better insights into its effect on diversity and population differentiation as measured by *FST*. In particular, they give a treatment of the interference selection regime, based on work by Good et al. (2014). The validity of the theory is tested against simulations. Since natural populations are often subdivided, such a theoretical advance is welcome.

The weakness relates to the realism of the assumptions used in the simulations and illustrations of the theoretical results. In the Methods (l.548-9), they say rather vaguely that their parameters are “biologically realistic on average”, but acknowledge that they were not trying to simulate real organisms. But how realistic are their parameters? They use a genome model of 104 neutral sites surrounded by two blocks of 350 selected sites. They don’t make it clear what this is supposed to represent; are these two small exons abutting a large intron, or a large intergenic region between two small exons? It certainly doesn’t represent even a single gene, and of course a lot of published data come from synonymous sites which are embedded in numerous selected sites.

Given that a typical gene has approximately 300 amino acids, our 700 selective sites is consistent with the typical number of sites in a coding region that may be

experiencing stronger selection. On the other hand, the large number of neutral sites we track are not recombining among each other; given the way SLiM tracks each new mutation as unique it doesn't matter whether these are tracked as belonging to the same "site" or different. With the 10^4 sites, we have plenty of replication of our measurements of π within each replicate gene to reduce measurement error. What is important is the total mutation rate for neutral variants, which is the parameter we use for comparison to theory here. We have edited the description of the genome in the paper to make these issues clearer.

Their total population size is 5000, and their non-zero recombination rate is *r* = 1.41x10-5. It's a good rule for simulations to ensure that the deterministic parameters scaled by effective population size are consistent with what you expect in a much larger natural population. They don't explicitly mention this, nor do they say what type of organism they have in mind. Let's assume that the total *Ne* is like that of a medium-sized mammal, 10^5 say. Then their *Nr* = 0.057 would correspond to *r* = 5.7 x 10-7; this is very high compared with a typical *r* of 10-8. Even an *N* of 106 gives an *r* that looks high.

We have two responses here. First, half of our simulations use *r* = 0, so we do consider low recombination rates. Second, as we discuss above, the intent of our paper is to investigate the accuracy of the approximations made by various theoretical approaches to this problem. The non-zero recombination rate we chose to use was effective for its intended purpose.

Similarly, their deleterious mutation *U* = 7 x 10-3 gives a per basepair mutation rate of 1 x 10-5; this corresponds to a mutation rate of 5 x 10-7 for *N* = 105 and 5 x 10-8 for *N* = 106, both rather high when compared with published estimates.

True. However, as we mention in response to the point above, in SLiM new mutations are attached to a location in the genome without overwriting other mutations. In SLiM one can consider the mutation rate per site as a mutation rate for a block of non-recombining sites. We have added a sentence to the Methods in the Simulation section to clarify this.

In addition, by using high mutation rates for deleterious alleles we can increase the magnitude of the effect and make comparisons to theory easier and more precise.

Similarly, it's not clear how their choice of selection coefficients correspond to the estimates obtained from population genomic studies, which suggest that the mean selection coefficient scaled by *Ne* is of the order of 10^3 or more, with a wide distribution around it. In the case of an island model, Wakeley (2003 Genetics 163:411) showed that the scaled selection parameters that control the distribution of allele frequencies correspond approximately to the product of selection coefficient and *Ne* for the whole population, total *N*/(1–*FST*), if subdivision is not too extreme. An *Nlocalt* of 15 would thus correspond to an *Net* of 15 with a neutral *FST* of 0.5 and

total $N=10^5$. Their strongest selection case is thus actually rather weak compared with the typical range of values suggested by the population genomics data.

We are not sure that the number used in the last two sentences match the values used in our paper. Our figures and simulations range up to a value of $N_{local}\ t$ = 15; given that we have 10 demes this means $N_{global}\ t$ = 150, or higher when divided by (1-Fst). However, we believe this was a typo in the reviewer's comments; our strongest selection has a Nt in the hundreds.

However, the theory works well for strong selection. The concerns that are the focus of this paper arise with weak and moderate selection, which we focus on here.

A weakness of most of the theoretical analyses of the interference selection limit is that they ignore reverse mutations, so they don't realistically predict the stochastic equilibrium situation that is likely to be reached with base substitutions; an exception is Devi et al (2023 Genetics 224: iyad091).

We agree that this is an excellent avenue for further work in general. However, it is beyond the scope of the present paper.

Finally, they don't say what they assume about the neutral mutation rate used to generate their diversity values. Looking at their Fig.3, these seem extremely high compared with most observed values for silent sites in natural populations. If they assumed the same mutation rate as for selected sites, 1 x 10-5, 4*Nu* would be 0.2, which presumably explains these high values, for which the usual infinite sites assumption used in the relating BGS predictions is somewhat inaccurate.

Please see above for our comments on the meaning in SLiM of this neutral mutation rate.

Overall, therefore, it's not clear that their results give a good guide as to what you might expect to see in population genomic data on real organisms.

We agree, but this is not the intent of the paper. Our intent is to test the efficacy of the new and old theory.

They strongly emphasise the importance of the interference selection limit, but the available data on the distributions of selection coefficients for deleterious mutations suggest that only a small fraction of mutations fall into this class (e.g., Kim, B et al 2017 Genetics 206:345). They may be important for the properties of low recombination rates of the genome, where *Ne* is strongly reduced by interference, but it's unlikely that this is true in many parts of most genomes. The statement on l.454-56,

that “most deleterious alleles are typically expected to be weakly deleterious and therefore suited to the interference selection regime” is thus misleading. In addition, in regions of the genome where recombination is low and interference is high, associative overdominance effects may also be operating, confounding some of the expectations under pure BGS; this possibility is not mentioned (Becher et al 2020 Curr Biol 30:94; Gilbert et al. 2020 Curr Biol 20:101).

We agree that the DFE of amino acid changing mutations has a much higher fraction of large effect mutations than the distribution we assume, although the Kim et al. reference above shows a substantial number of coding-region mutations with weak effects. However, less is known about the DFE of non-coding changes. Given our collective ignorance on this, it is useful to have an effective theory that works in this weaker-selection regime.

We now cite the Kim et al. 2017 paper in the introduction (line 183) and we have removed the reference to this issue in the discussion.

Specific comments

l.60 Qualify by saying that they have to be sufficiently strongly selected.

With high migration rates, as this sentence is discussing, the amelioration of the background selection effect takes place across all values of the selection coefficient (see Figure 1A&B.)

l.72 The new paper by Buffalo & Kern should be cited in a revision (2024, PloS Genet 20:e1011144). This uses an approach to predicting B with weak selection based on Santiago & Caballero 2016 (Genetics 204: 1267), which is less impenetrable than that of Good et al.

We agree. We have re-written the main body of the paper in terms of the Buffalo and Kern derivation, while leaving the information about the Good et al. approach in an appendix.

l.73 Recent work on this by Parul Johri and others should be cited.

Thanks. We’ve added this reference in a few places.

l.78 It would be more accurate to say that the modelling of BGS involves pairwise coalescent times; this accurately predictsthe π values under the infinite sites assumption (not met accurately in the present simulations).

Yes, we agree about the strict meaning of B and have conditioned our statement here to refer to the infinite sites model. However, this does not affect the match of the theory to the simulations. In SLiM, all new mutations are attached to the old genotype and don't overwrite other mutations that are present at the same site. So SLiM effectively is modeling an infinite sites model. We have made this more clear in the Methods in the Simulations section in the revision.

l.86 The 1st application of this idea to BGS was in ref. 3.

We think the essence of this idea is in the Santiago and Caballero 1995 paper, which we cite here.

l.96 This is only one definition of *FST*; others are frequently used in relation to population genomics data, as is mentioned much later.

Agreed, but this quantity is estimated by Weir and Cockerham's theta, which is by far the most widely used estimator of F_{ST}. We add comment to this effect on line 106.

l.103 It's odd not to mention ref. 32 here, which drew attention to this explanation for high
FST in low recombination regions of Drosophila.

Agreed. We've added that reference here.

l.116 Again, the effect of weak selection was recognized much earlier than ref. 29 (see ref. 34).

We credit ref. 29 for defining an 'interference selection' and 'background selection regime'. We have added a reference to Charlesworth 2012 here to direct the reader to previous work.

l.138 There's a follow-up paper (Melissa et al. 2022 Genetics 221:iyac053), which modifies the conclusions somewhat (I don't know how important this is).

Melissa et al. 2022 extend the model of Good et al. (2014) to the regime of scaled selection coefficients that are near one. However, it is unclear to us how to generate prediction of *B* given the findings of their paper due to the primary application of their model being to generate accurate predictions for the site frequency spectrum under a distribution of both beneficial and deleterious selection coefficients.

l.159 Ref. 7 is very old for this topic; see my general comments.

We have now added reference to Kim et al 2017 as suggested by this reviewer.

l.180 What about low recombination regions, which can be quite large in many organisms? There's increasing evidence that *FST* is elevated in such regions, e.g. Burri et al. 2015 Genome Res. 25**:** 1656.

Even with no recombination, there is little effect on F_{ST} of background selection when the migration rate is high enough (see Figure 2). The Burri et al. case deals with F_{ST} values in the 0.1 to 0.2 range, reflecting an example where the migration rate is not high, and in this case our theory does predict an effect of BGS on F_{ST}. (This sentence in our manuscript limits itself to cases with non-trivial migration.)

Because we use large mutation rates in our simulations to make the difference in the effects more clear, we are effectively modeling a region of several hundred coding regions within a non recombining block when we set the recombination rate to 0. This should effectively model the effects on F_{ST} of a large segment of non-recombining DNA.

l.253 The justification for this relation should be mentioned.

This section is now in the supplemental text; we have added a reference for this relation there.

l.285**-**286 Is this really very important- if all forces are weak, it really shouldn't matter. If migration is strong, then subdivision is not important.

This derivation provides an accurate representation of F_{ST} given the order of events of our simulations, and is therefore important to our results, which otherwise have discrepancies caused by order of events and not the accuracy of accounting for background selection.

l.325 This is presumably a reflection of the same effect observed for diversity, e.g. by Gordo et al. 2002 Genetics 78: 835.

Yes we agree, this is due to the pattern of effect on B in both cases. We now refer to the Gordo et al paper here.

l.345 The usual term is 'scaled selection coefficients'.

Fixed.

l.352 This effect was already mentioned in ref. 1.

We add to the start of the sentence that this was indeed the expectation. The intention of this paragraph was not to provide a novel result, but to orient the reader.
