# Supplementary material for: FST and genetic diversity in an island model with background selection
Source: PLoS Genet. 2024 Dec 2;20(12):e1011225. doi: 10.1371/journal.pgen.1011225 (PMC11637402; doi:10.1371/journal.pgen.1011225)
Supplement: Attachment — Submitted filename: Hasan-Whitlock-R1.pdf [file pgen.1011225.s008.pdf]

The paper has been substantially revised and made more intelligible by refocussing the theoretical work on the methods based on Santiago & Caballero's generalization of Robertson's heritable variance in fitness approach to the effects of selection on N_e. I still have a number of critical comments, however.

They give a rather misleading impression of the history of work on background selection and interference selection, which should probably be corrected. The failure of the strong selection formula with weak selection was already noted in ref. 1. It was investigated in some detail for the non-recombination case by Charlesworth et al in connection with distortions of gene geneaologies (1995, Genetics 141:1619) and then by ref. 45, long before the work by Desai's group. The term "interference selection" was invented by Comeron & Kreitman (2000 Genetics 161:389), not by ref. 20; it's certainly a better term than the original "Weak selection Hill-Robertson interference" of ref. 27. These two papers are not cited. Furthermore, ref. 23 did not model background selection; it assumed no linkage and used a directional selection perspective; Ref. 3 was the first to apply Robertson's heritable variance approach to background selection. They could actually save trouble for the reader by referring early on to a recent review of this topic, e.g. Charlesworth & Jensen 2021 Ann Rev Ecol Evol Syst 52:177.

They also did not respond to my comment about associative overdominance and its effects on diversity in low recombination regions, for which evidence has been provided by Becher et al (2020) and Gilbert et al. (2020); if we are supposed to take interference selection seriously, this feature of it cannot simply be ignored, although it's probably not an issue except for low recombination rate.

They also fail to cite Devi et al. (2023), which provides a different perspective on interference selection.

The specific comments below point out some remaining problems; in general, they haven't really addressed the criticism that their results are not based on a realistic gene model or mutation rates etc, so it is hard to relate them to empirical results. The authors have responded by simply saying that they want to lay out an improved theory of background selection in a structured population and I think they have largely succeeded in this.

It's an editorial decision as to whether this is the type of paper that the journal want to publish, if the remaining technical points can be satisfactorily dealt with.

l.154 'fixation' is better than 'substitution'.

l.274 It's not clear how you can simply use an expression for fixation probability that uses the free recombination formula when there selective interference; this seems like a self-contradiction. I don't think this problem was satisfactorily explored in the cited papers; it problem should at least be commented on.

l.279 R should be in italics.

l.310-313 This comment will be rather impenetrable to most readers; the point is that there are two N_e's, one that deals with fixation probabilities, and the other that deals with diversity, leading to two different *B* measures; in general, *B* for diversity is bigger than that for fixation. It would be helpful to make this clear and to explain that the second B is the appropriate one for this analysis.

l.349 State that semi-dominance is assumed (I presume that this is the case).

l.533 Also ref. 32.

l.597-599 I am still not convinced that their mutation and recombination rates are biologically realistic. As I commented before, if you rescale their deleterious mutation rate per selected site (1×10^{-5}) to a population size of 10^5, it becomes 5×10^{-7}, well over an order of magnitude higher than measured rates for most multicells. A similar remark applies to the higher recombination rate that they use. The two work in opposite directions, of course. Their gene model also seems arbitrary and unrealistic; it is presumably supposed to represent a large intron in between two exons, but they don't explain or justify this choice.

l.609 I am not familiar with the details of SLiM, so I found this explanation hard to follow. The point about the infinite sites model is that at most one mutation is segregating at a single nucleotide site. Thus, their statement that "a novel mutation at a polymorphic site simply acts as a novel allele" doesn't seem to match the infinite sites model; rather, it seems to correspond to the infinite alleles model, which has somewhat different properties. What happens if two mutations segregate at the same site? It's thus not clear to me that they have satisfactorily dealt with the query that I raised concerning their diversity values. My understanding is that SLiM now uses gene trees to generate diversity values, so could't the pairwise coalescent times from these trees have been used?
