# Supplementary material for: FST and genetic diversity in an island model with background selection
Source: PLoS Genet. 2024 Dec 2;20(12):e1011225. doi: 10.1371/journal.pgen.1011225 (PMC11637402; doi:10.1371/journal.pgen.1011225)
Supplement: Attachment — Submitted filename: response_to_reviewer_3.pdf [file pgen.1011225.s009.pdf]

In this document, we provide a response to reviewer 3 as well as where we made changes to the manuscript in response to reviewer 3's comments. These changes are marked in a blue font on the 'marked' copy of the manuscript included in the submission.

The paper has been substantially revised and made more intelligible by refocusing the theoretical work on the methods based on Santiago & Caballero's generalization of Robertson's heritable variance in fitness approach to the effects of selection on Ne. I still have a number of critical comments, however.

They give a rather misleading impression of the history of work on background selection and interference selection, which should probably be corrected. The failure of the strong selection formula with weak selection was already noted in ref. 1. It was investigated in some detail for the non-recombination case by Charlesworth et al in connection with distortions of gene genealogies (1995, Genetics 141:1619) and then by ref. 45, long before the work by Desai's group. The term "interference selection" was invented by Comeron & Kreitman (2000 Genetics 161:389), not by ref. 20; it's certainly a better term than the original "Weak selection Hill-Robertson interference" of ref. 27. These two papers are not cited.

We have now modified our introduction of the history of theoretical work on background selection and interference in Lines 123–162 to make it clear to the reader that the importance of the assumption of the strength of selection in these classic models was indeed already known by its authors. We have also now given reference to Charlesworth et al. (1995) and Comeron & Kreitman (2000) in this section.

Furthermore, ref. 23 did not model background selection; it assumed no linkage and used a directional selection perspective;

Ref. 23 (Santiago & Caballero 2016) computed fixation effective sizes under a background selection model with weak selection (see Fig. 1) . They account for linkage using the L term (map length) in Eq. 2&3. When T=0, their Eq. 3 simplifies to the prediction of B of the background selection model of Hudson & Kaplan (1995).

Ref. 3 was the first to apply Robertson's heritable variance approach to background selection. They could actually save trouble for the reader by referring early on to a recent review of this topic, e.g. Charlesworth & Jensen 2021 AnnRev Ecol Evol Syst 52:177.

Good point. We have now included reference to Charlesworth & Jensen 2021 early on in the introduction (Lines 67, 87) to assist the reader.

They also did not respond to my comment about associative overdominance and its effects on diversity in low recombination regions, for which evidence has been provided by Becheret al (2020) and Gilbert et al. (2020); if we are supposed to take interference selection seriously, this feature of it cannot simply be ignored, although it's probably not an issue except for low recombination rate.

We agree that associative overdominance is an important empirical phenomenon to consider under low recombination in the interference selection regime. We have now added mention of it in Lines 579–583 in the discussion section (including reference to these two articles), describing how it may emerge when dealing with recessive deleterious alleles in this regime. However, AOD is not of strong relevance to the case of semi-dominant deleterious alleles we treat in this manuscript.

They also fail to cite Devi et al. (2023), which provides a different perspective on interference selection.

We have now added reference to Devi et al. (2023) on Line 149, where we describe work on the *interference selection regime*.

The specific comments below point out some remaining problems; in general, they haven't really addressed the criticism that their results are not based on a realistic gene model or mutation rates etc, so it is hard to relate them to empirical results. The authors have responded by simply saying that they want to lay out an improved theory of background selection in a structured population and I think they have largely succeeded in this.It's an editorial decision as to whether this is the type of paper that the journal want to publish, if the remaining technical points can be satisfactorily dealt with.

l.154 'fixation' is better than 'substitution'.

Done

l.274 It's not clear how you can simply use an expression for fixation probability that uses the free recombination formula when there selective interference; this seems like a self contradiction. I don't think this problem was satisfactorily explored in the cited papers; it problem should at least be commented on.

The formula does account for the effects of limited recombination. These effects are captured by the Q_i^2 term. See line 259

l.279 R should be in italics.
Fixed, thanks

l.310-313 This comment will be rather impenetrable to most readers; the point is that there are two Ne's, one that deals with fixation probabilities, and the other that deals with diversity, leading to two different B measures; in general, B for diversity is bigger than that for fixation. It would be helpful to make this clear and to explain that the second B is the appropriate one for this analysis.

Good point. We've now added a few sentences to clarify this point and make the distinction between the two Ne's on lines 311–321

l.349 State that semi-dominance is assumed (I presume that this is the case).

Yes, we have clarified this in line 358–359

l.533 Also ref. 32.

Good point. We have now cited ref. 32 here (now reference 35).

l.597-599 I am still not convinced that their mutation and recombination rates are biologically realistic. As I commented before, if you rescale their deleterious mutation rate per selected site (1 x 10–5) to a population size of 10^5, it becomes 5 x 10^7, well over an order of magnitude higher than measured rates for most multicells. A similar remark applies to the higher recombination rate that they use. The two work in opposite directions, of course.Their gene model also seems arbitrary and unrealistic; it is presumably supposed to represent a large intron in between two exons, but they don't explain or justify this choice.

Our genetic architecture was chosen to be able to test the accuracy of the theory. Our parameters were not intended to reflect any particular gene model. We used a high mutation rate so that the effects of background selection would be distinguishable between different theoretical predictions, particularly for the weak selection case where otherwise the predicted effects are indistinguishable due to measurement error. To be quite frank, we don't consider our simulated region a single gene, and we do not claim it is in the paper. Having said that, the number of deleterious mutations per centiMorgan that we use here is biologically relevant.

l.609 I am not familiar with the details of SLiM, so I found this explanation hard to follow. The point about the infinite sites model is that at most one mutation is segregating at a single nucleotide site. Thus, their statement that “a novel mutation at a polymorphic site simply acts as a novel allele” doesn’t seem to match the infinite sites model; rather, it seems to correspond to the infinite alleles model, which has somewhat different properties. What happens if two mutations segregate at the same site?

SLiM indeed uses an infinite allele model. We’ve now removed our sentence in the discussion where we state that SLiM uses an infinite sites model to reflect this.

It’s thus not clear to me that they have satisfactorily dealt with the query that I raised concerning their diversity values. My understanding is that SLiM now uses gene trees to generate diversity values, so could’t the pairwise coalescent times from these trees have been used?

As we said in our initial response, the large number of neutral sites in our simulations is not intended to reflect a gene model, but to minimize our measurement error in estimating π. In any case, note that our mutation rate at a neutral site gives $N\mu < 0.1$, meaning our theoretical predictions of π (Line 688) should be accurate.
